# Synergistic anticancer effects of a bioactive subfraction of *Strobilanthes crispus* and tamoxifen on MCF-7 and MDA-MB-231 human breast cancer cell lines

**DOI:** 10.1186/1472-6882-14-252

**Published:** 2014-07-18

**Authors:** Nik Soriani Yaacob, Nik Nursyazni Nik Mohamed Kamal, Mohd Nor Norazmi

**Affiliations:** 1 Department of Chemical Pathology, School of Medical Sciences, Universiti Sains Malaysia Health Campus, 16150 Kubang Kerian, Kelantan, Malaysia; 2 School of Health Sciences, Universiti Sains Malaysia Health Campus, 16150 Kubang Kerian, Kelantan, Malaysia

**Keywords:** *Strobilanthes crispus*, Tamoxifen, Breast cancer, Synergism, Apoptosis, Cytotoxicity, Mitochondrial membrane potential, Caspase

## Abstract

**Background:**

Development of tumour resistance to chemotherapeutic drugs and concerns over their toxic effects has led to the increased use of medicinal herbs or natural products by cancer patients. *Strobilanthes crispus* is a traditional remedy for many ailments including cancer. Its purported anticancer effects have led to the commercialization of the plant leaves as medicinal herbal tea, although the scientific basis for its use has not been established. We previously reported that a bioactive subfraction of *Strobilanthes crispus* leaves (SCS) exhibit potent cytotoxic activity against human breast cancer cell lines. The current study investigates the effect of this subfraction on cell death activities induced by the antiestrogen drug, tamoxifen, in estrogen receptor-responsive and nonresponsive breast cancer cells.

**Methods:**

Cytotoxic activity of SCS and tamoxifen in MCF-7 and MDA-MB-231 human breast cancer cells was determined using lactate dehydrogenase release assay and synergism was evaluated using the CalcuSyn software. Apoptosis was quantified by flow cytometry following Annexin V and propidium iodide staining. Cells were also stained with JC-1 dye to determine changes in the mitochondrial membrane potential. Fluorescence imaging using FAM-FLICA assay detects caspase-8 and caspase-9 activities. DNA damage in the non-malignant breast epithelial cell line, MCF-10A, was evaluated using Comet assay.

**Results:**

The combined SCS and tamoxifen treatment displayed strong synergistic inhibition of MCF-7 and MDA-MB-231 cell growth at low doses of the antiestrogen. SCS further promoted the tamoxifen-induced apoptosis that was associated with modulation of mitochondrial membrane potential and activation of caspase-8 and caspase-9, suggesting the involvement of intrinsic and extrinsic signaling pathways. Interestingly, the non-malignant MCF-10A cells displayed no cytotoxicity or DNA damage when treated with either SCS or SCS-tamoxifen combination.

**Conclusions:**

The combined use of SCS and lower tamoxifen dose could potentially reduce the side effects/toxicity of the drug. However, further studies are needed to determine the effectiveness and safety of the combination treatment *in vivo*.

## Background

Tamoxifen is the gold standard hormonal therapy for estrogen receptor-positive (ER+) breast cancers by acting as an estrogen antagonist on breast tissue [1]. It is also used as adjuvant therapy for breast cancer to reduce the risk of recurrence [2]. However, about half of these cancers do not respond to tamoxifen. Another important drawback of the drug is its reduced efficacy with long-term use, as most tumours eventually develop resistance to tamoxifen [3,4]. In addition, adverse side effects including uterine cancer and thromboembolic disease have been attributed to the use of tamoxifen [1]. In fact, concerns over toxicity of chemotherapeutic drugs have primarily contributed to the increased use of herbal and natural products for cancer treatment [5]. The combined use of natural products and conventional anticancer drugs is believed to enhance the efficacy of anticancer treatment due to their potential additive or synergistic effects. Furthermore, such combination treatment could potentially reduce the side effects of chemotherapy. However, such purported advantages of natural products have not been scientifically established.

The flowering shrub, *Strobilanthes crispus* (Acanthacea) is traditionally used as a folklore medicinal plant in Malaysia and Indonesia. Its leaves have been traditionally used to treat various ailments including breast and uterine cancers and gastrointestinal and kidney diseases [6,7]. The plant is locally known as ‘pecah beling’ or ‘kecibeling’ in Indonesia and ‘pecah kaca’ or ‘jin batu’ by the Malays, or as ‘bayam karang’ by the Orang Asli tribe in Malaysia. The leaves of this plant taken orally, is believed to enhance the immune system [8]. The plant is also known as Hei Mian Jiang Jun (Black-faced General) to the local Chinese community and the leaves of the plants are normally boiled and taken as tea or is mixed with other herbs. Based on its traditional use, the plant is also commercialised as tea and dehydrated herb, and recommended for those with cancer and other ailments. Its consumption as herbal tea also provides additional antioxidants such as catechins [9]. The water extract of *S. crispus* contains compounds that could block the proliferation of retroviruses in T-cell leukemia by attaching to the active sites of reverse transcriptase [10]. Cytotoxicity against liver, colon and breast cancer cell lines [11] and chemopreventive effects of *S. crispus* extract against rodent hepatocellular carcinoma [12] have been reported. Findings from our research group showed that a bioactive subfraction of *S. crispus* extract induced apoptosis of breast and prostate cancer cells [13]. The current study further investigates the cellular anticancer activities of an *S. crispus* subfraction (SCS) and its ability to modulate tamoxifen-induced effects on the breast cancer cell lines, MCF-7 and MDA-MB-231, as well as the non-cancerous breast epithelial cell line, MCF-10A.

## Methods

### Chemicals

Dulbecco modified Eagle’s medium (DMEM), Ham F12-K medium, Rosselle’s Park Memorial Institute medium (RPMI-1640) and penicillin/streptomycin/glutamine (100×) were purchased from GIBCO BRL (UK). Fetal bovine serum (FBS) and trypsin/EDTA were purchased from Hyclone (USA) and BDH Chemicals (UK), respectively. Dimethyl sulphoxide (DMSO), hydrogen peroxide (H_2_O_2_) and tamoxifen were purchased from Sigma-Aldrich (USA). Phosphate buffered saline (PBS) was purchased from Amresco (USA). All chemicals used in the experiments were of analytical grade.

### Plant material

The *S. crispus* plants were collected from Tasek Gelugor, Pulau Pinang, Malaysia and authenticated by Mr Baharuddin Sulaiman, a taxonomist at the School of Biological Sciences, Universiti Sains Malaysia. A voucher specimen of the plant (no. 11046), was then prepared and deposited at the herbarium of the School of Biological Sciences [13].

### Cell culture and treatment

MCF-7 and MDA-MB-231 cells (American Type Culture Collection, Rockville, USA) were grown in RPMI-1640 and Dulbecco’s modified Eagle’s medium (DMEM), respectively, supplemented with 10% fetal bovine serum (Hyclone, USA) and 100 units/ml penicillin. MCF-10A cells were maintained in DMEM/F12 complete growth medium. All cells were maintained at 37°C in a humidified condition with 5% CO_2_. Prior to treatment, the culture medium was replaced with medium supplemented with 2% fetal bovine serum. Tamoxifen (Sigma Aldrich, USA) was freshly diluted in the culture medium for each experiment. The SCS was prepared as previously reported (previously coded as SC/D-F9) [13] and stored as crystalline solids at -80°C until use.

### Determination of cytotoxic effects and combination index

MCF-7 and MDA-MB-231 cells were seeded in 24-well plates (Corning, Baxter Scientific, McGaw Park, IL) at 100,000 cells per ml, allowed to attach overnight and treated with SCS (8.5 and 15.0 μg/ml [13]), tamoxifen (2.5-15 μM) or their combination for up to 48 h at 37°C. Control cells received the vehicle, dimethyl sulfoxide (<0.1%). The cytotoxic effect was determined using lactate dehydrogenase (LDH)-release assay (Roche Diagnostics, Mannheim, Germany) as described by the manufacturer. The combined effect of SCS and tamoxifen was then analysed using the CalcuSyn software using non-constant ratio combination design (Biosoft, UK). All tests were performed in triplicates.

### Assessment of apoptosis and necrosis

Apoptotic or necrotic cell death was quantified by flow cytometry using the fluorescein isothiocyanate (FITC)-labelled annexin V and propidium iodide (PI) (Annexin-V-FLUOS Staining Kit [Roche, Germany]) according to the manufacturer’s recommendations. Cell nuclei were stained with Hoechst dye. Cells were harvested after 24 and 48 h incubation with SCS and tamoxifen by using 0.02% trypsin-EDTA, pelleted by centrifugation at 1,000 rpm and washed in phosphate buffered saline. A minimum of 10,000 events were collected and analysed using the FACS Calibur instrument and CellQuest Pro software (Becton Dickinson, USA).

### Assessment of changes in the mitochondrial membrane potential

Changes in the mitochondrial membrane potential (∆Ψ_m_) were determined quantitatively by flow cytometry, using 5,5′,6,6′-tetrachloro-1,1′,3,3′-tetraethyl-benzimidazolylcarbocyanine iodide (JC-1) dye (Invitrogen, USA) at 24 and 48 h post-treatment according to the manufacturer’s instructions. JC-1 accumulates within the intact mitochondria to form multimer J-aggregates that resulted in a change of fluorescence from red to green indicating decreased ∆Ψ_m_. The green and red fluorescence was estimated in FL1 and FL2 channel, respectively, using a minimum of 10,000 events. The percentage of cells with green fluorescence (JC-1 monomers) which represent depolarized cells was measured.

### Assessment of caspase 8 and caspase 9 activities

Cells were cultured on chamber slides and treated with SCS and tamoxifen for 24 h. FAM-FLICA™ Caspase-8 and FAM-FLICA™ Caspase-9 Assay kits (Immunochemistry Technologies) were used to detect caspase 8 and caspase 9 activation in the cells, respectively. This methodology is based on the use of non-cytotoxic and cell-permeable fluorochrome inhibitor of caspases (FLICA) that binds covalently to active caspases and emits green fluorescence. Cells with activated caspase 8 or caspase 9 were observed using the fluorescence microscope according to the manufacturer’s guidelines.

### Assessment of DNA damage

MCF-10A cells cultured in 25 cm^2^ flasks were treated with SCS (8.5 μg/ml) and tamoxifen (5 μM) alone or in combination for 24 h. DMSO (0.1%) was used as a vehicle control and hydrogen peroxide (H_2_O_2_) was used as a positive control (20 min incubation). DNA damage was evaluated using the Comet assay (Trevigen, USA) following the manufacturer’s instructions.

### Statistical analyses

At least three independent experiments were carried out. Significant differences in the mean values were calculated by one-way analysis of variance (ANOVA) followed by post hoc Tukey multiple comparison test using IBM® SPSS® Statistics 20 (United States) for Windows and considered statistically significant at p < 0.05.

## Results

### Synergistic cytotoxicity of SCS and tamoxifen

**Figure 1:**
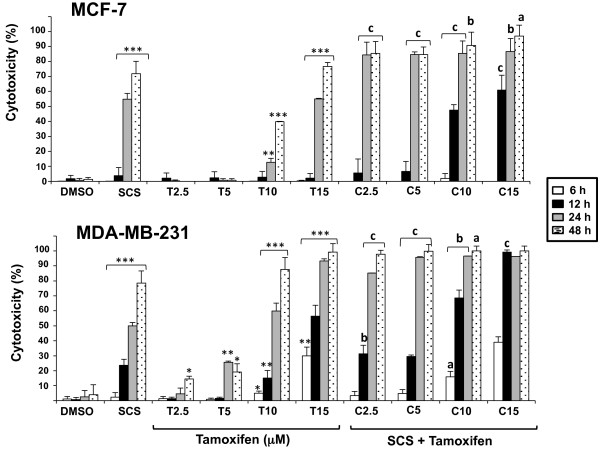
**Enhancement of tamoxifen-induced cytotoxicity in breast cancer cell lines by SCS.** Percentage of cell death following treatment with DMSO (control); SCS (8.5 and 10.0 μg/ml for MCF-7 and MDA-MB-231 cells, respectively); 2.5, 5.0, 10.0 and 15.0 μM tamoxifen (T2.5-T15); and their combination with SCS (C2.5-C15) was determined at 6, 12, 24 and 48 h using the LDH assay. Data shown are the mean values ± SD from three independent experiments. Statistical analyses was determined using one-way ANOVA followed by post hoc Tukey multiple comparison test with *p < 0.05, **p < 0.01, ***p < 0.001, significantly different from control; ^a^p < 0.05, ^b^p < 0.01, ^c^p < 0.001, combination treatment compared to tamoxifen.

Tamoxifen showed a dose-dependent cytotoxicity on MCF-7 and MDA-MB-231 cells (Figure 1). At 24 h, about 60% and 90% cell death were recorded with 15 μM tamoxifen, respectively. Efficacy of treatment was increased with the combination of SCS and tamoxifen compared to either single agent. The presence of SCS (8.5 μg/ml or 10.0 μg/ml) further increased the percentage of cell death induced by tamoxifen in both cell lines. When given alone, no significant cell death was recorded with tamoxifen treatment at the low concentrations of 2.5 and 5 μM in MCF-7 cells but 90% cell death was recorded with the combined treatment of tamoxifen at these concentrations and SCS after 24 h (p <0.001). Similar cell death promoting effect was observed in MDA-MB-231 cells with 85% and 95% cell death obtained with 2.5 and 5 μM tamoxifen and SCS combination at 24 h (p < 0.001). The promoting effect of SCS was time-dependent as well as tamoxifen dose-dependent in both cell lines and could be observed as early as 12 h post treatment. Maximum cell death was achieved with all combined treatment groups at 48 h.

**Figure 2:**
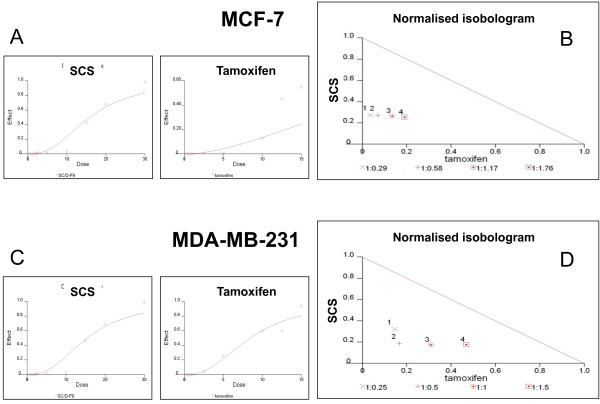
**Dose-effect analysis and the combination effect of tamoxifen and SCS.** Dose-effect analysis was performed using 8.5-12.5 μg/ml SCS and 2.5-15 μM tamoxifen, for 24 h. Dose-effect curve of each agent alone for MCF-7 cells **(A)** and MDA-MB-231 cells **(C)** is representative of three independent experiments. EC_50_ concentrations of SCS and 2.5-15 μM tamoxifen were used for combination effect analysis using the CalcuSyn program and normalized isobolograms for MCF-7 **(B)** and MDA-MB-231 **(D)** cells are shown. Diagonal line is the additivity line and data points below this line correspond to a synergistic effect. The concentration ratios between SCS and tamoxifen are indicated below the isobologram.

Dose-effect analysis of the combination treatment was carried out based on the method of Chou-Talalay and the type of interaction between the two agents was evaluated using the combination index (CI) [14,15]. According to this method, synergism is indicated by a CI of less than 1, additivity by a CI equal to 1, and antagonism by a CI greater than 1. Normalised isobolograms constructed for non-constant ratio drug combinations (Figure 2) display data points below the additivity line, indicating strong synergy in growth inhibition of MCF-7 and MDA-MB-231 cells by SCS (8.5 μg/ml or 10.0 μg/ml) and tamoxifen (2.5 to 15 μM) combination. The calculated CI values obtained were 0.32 – 0.40 for MCF-7 cells and 0.29 – 0.52 for MDA-MB-231 cells at 84 – 97% fractions affected. Since SCS is found to significantly promote the growth-inhibitory effect of tamoxifen at low doses, 2.5 and 5 μM tamoxifen were therefore used for subsequent treatments.

### Promotion of tamoxifen-induced apoptosis by SCS

**Figure 3:**
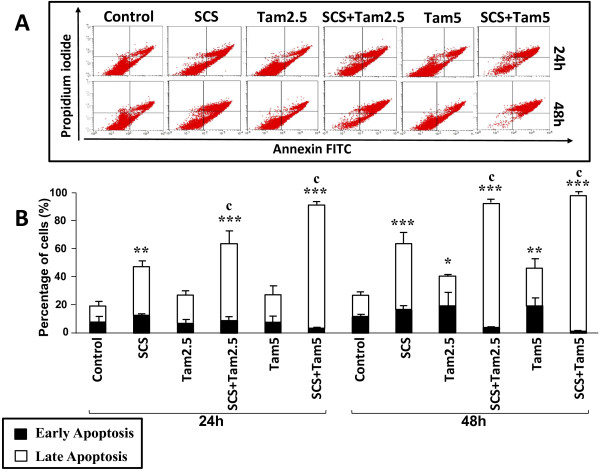
**Promotion of tamoxifen-induced apoptosis of MCF-7 cells by SCS.** Cells were treated with 8.5 μg/ml SCS and 2.5 (Tam2.5) or 5.0 μM tamoxifen (Tam5) alone and in combination for 24 and 48 h. Harvested cells were incubated with annexin-V antibody and PI dye and analysed by flow cytometry. **(A)** Quadrant location for the representative dot plots: lower left – negative immunofluorescence (living cells); lower right – annexin V positive (early apoptosis); upper left – PI positive (necrosis), upper right – annexin V and PI positive (late apoptosis). **(B)** Each bar represents mean ± SD of three independent experiments. *p < 0.05, **p < 0.01, ***p < 0.001 significantly different from control; ^c^p < 0.001 SCS + Tam vs Tam alone (one-way ANOVA and post hoc Tukey multiple comparison test).

**Figure 4:**
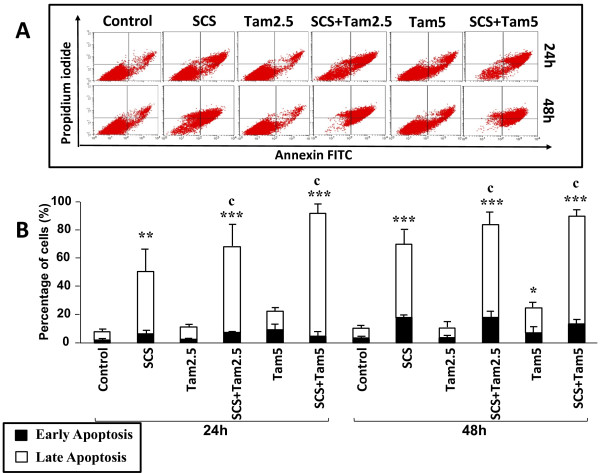
**Promotion of tamoxifen-induced apoptosis of MDA-MB-231 cells by SCS.** Cells were treated with 10.0 μg/ml SCS and 2.5 (Tam2.5) or 5.0 μM tamoxifen (Tam5) alone and in combination for 24 and 48 h. Harvested cells were incubated with Annexin-V antibody and PI dye and analysed by flow cytometry. **(A)** Quadrant location for the representative dot plots: lower left – negative immunofluorescence (living cells); lower right – annexin V positive (early apoptosis); upper left – PI positive (necrosis), upper right – annexin V and PI positive (late apoptosis). **(B)** Each bar represents mean ± SD of three independent experiments. *p < 0.05, **p < 0.01, ***p < 0.001 significantly different from control; ^c^p < 0.001 SCS + Tam vs Tam alone (one-way ANOVA and post hoc Tukey multiple comparison test).

Phosphatidylserine is translocated from the inner membrane to the outer layer during early stages of apoptosis and could be detected using the phospholipid-binding protein, annexin V. Annexin V and PI double staining could discriminate between apoptotic and necrotic cells. We have previously shown that SCS is capable of inducing apoptosis. Here, flow cytometric analysis showed that the combination of SCS and tamoxifen resulted in promotion of apoptosis with a significant increase in late-stage apoptosis of MCF-7 cells compared to either treatment alone (p < 0.001; Figure 3). The combination of 2.5 μM tamoxifen and SCS caused 55% late apoptosis after 24 h treatment which increased to 88% at 48 h, resulting in total apoptosis of 63% and 92%, respectively. On the other hand, only 27% and 40% apoptosis was seen with tamoxifen alone at 24 h and 48 h, respectively. The combination of 5.0 μM tamoxifen and SCS resulted in 92% and 98% total apoptosis at 24 h and 48 h, respectively. Not more than 2% necrotic cells were observed with all treatments.About 11% apoptotic MDA-MB-231 cells were observed following treatment with 2.5 μM tamoxifen while 5.0 μM tamoxifen induced up to 25% apoptosis (Figure 4). The percentage of cell death increased significantly with co-administration of SCS (p < 0.001). Total apoptosis of 68% - 84% was recorded with 2.5 μM tamoxifen-SCS combination and more than 90% with 5.0 μM tamoxifen-SCS combination. The induction of apoptosis was time- and dose-dependent in both cells and the 4 to 9-fold increase in the number of cells at late stage apoptosis indicates that SCS not only promoted apoptotic cell death but also enhanced the rate of apoptosis.

### Enhancement of mitochondrial membrane depolarization by SCS and tamoxifen

**Figure 5:**
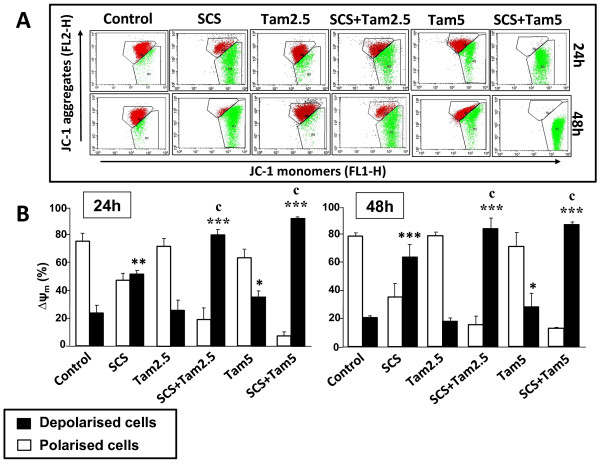
**Depolarization of the mitochondrial membrane of MCF-7 cells by SCS alone and in combination with tamoxifen.** MCF-7 cells treated with 8.5 μg/ml SCS and 2.5 (Tam2.5) or 5.0 μM tamoxifen (Tam5) alone and in combination for 24 and 48 h were incubated with the JC-1 dye and analysed by flow cytometry. **(A)** Representative dot plots: J-aggregated red fluorescence (polarized cells); JC-1 monomer green fluorescence (depolarized cells). **(B)** Each bar represents mean ± SD of three independent experiments. *p < 0.05, **p < 0.01, ***p < 0.001 significantly different from control; ^c^p < 0.001 SCS + Tam vs Tam alone (one-way ANOVA and post hoc Tukey multiple comparison test).

**Figure 6:**
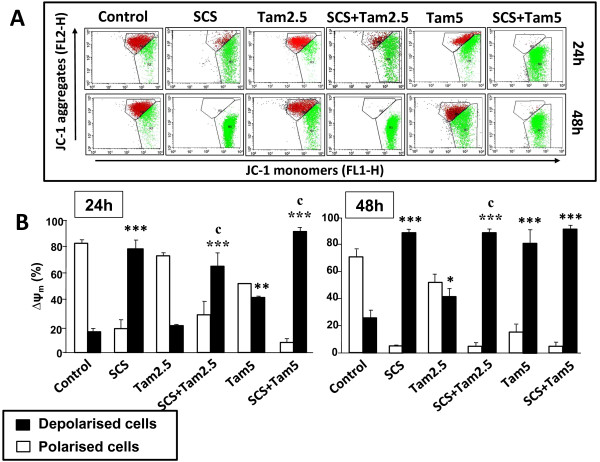
**Depolarization of the mitochondrial membrane of MDA-MB-231 cells by SCS alone and in combination with tamoxifen.** MDA-MB-231 cells treated with 10.0 μg/ml SCS and 2.5 (Tam2.5) or 5.0 μM tamoxifen (Tam5) alone and in combination for 24 and 48 h were incubated with the JC-1 dye and analysed by flow cytometry. **(A)** Representative dot plots: J-aggregated red fluorescence (polarized cells); JC-1 monomer green fluorescence (depolarized cells). **(B)** Each bar represents mean ± SD of three independent experiments. *p < 0.05, **p < 0.01, ***p < 0.001 significantly different from control; ^c^p < 0.001 SCS + Tam vs Tam alone (one-way ANOVA and post hoc Tukey multiple comparison test).

A decrease in the ∆ψ_m_ is considered as one of the earliest events in apoptosis. Figures 5 and 6 show that SCS significantly induced mitochondrial membrane depolarization in both MCF-7 (50% at 24 h [p < 0.01]; 69% at 48 h [p < 0.001]) and MDA-MB-231 (77% at 24 h; 89% at 48 h [both p < 0.001]) cells. On the other hand, 2.5 μM tamoxifen had no significant effect while 5.0 μM tamoxifen produced 30 to 35% depolarized MCF-7 cells (Figure 5). Enhanced alteration in the ∆ψ_m_ occured in the presence of both tamoxifen and SCS compared to tamoxifen alone (p < 0.001) whereby the combination treatment caused up to 90% cells to be depolarized. Significantly higher percentages of MDA-MB-231 cells were also depolarized when treated with both tamoxifen and SCS compared to tamoxifen alone (p < 0.001) but these were attributed to the potent effect of SCS itself (Figure 6). These results suggest the involvement of the mitochondrial pathway in SCS-induced cell death either alone or in combination with tamoxifen in both cell lines.

### SCS and tamoxifen activate caspase 8 and caspase 9

**Figure 7:**
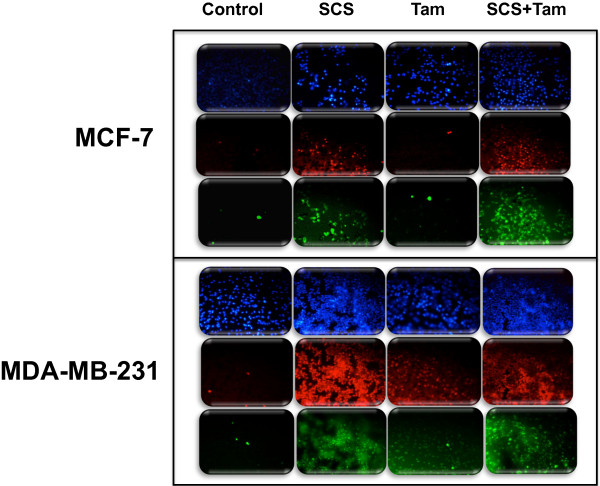
**Activation of caspase 8 in MCF-7 and MDA-MB-231 cells by SCS alone and in combination with tamoxifen.** Cells were stained with FLICA™ FAM-VAD-FMK for detection of active caspase-8 and labeled with PI and Hoescht stain following treatment with SCS (8.5 and 10.0 μg/ml for MCF-7 and MDA-MB-231 cells, respectively), tamoxifen (5.0 μM) or their combination for 24 h. Caspase-FLICA stains active caspases green; PI stains dead or dying cells red; Hoescht stains the nuclei of all cells blue.

**Figure 8:**
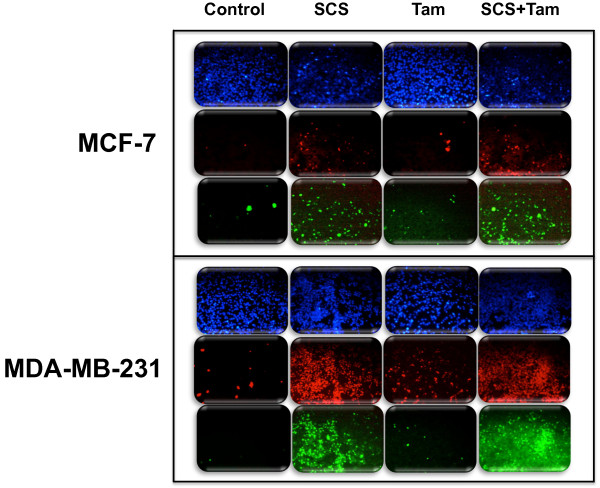
**Activation of caspase 9 in MCF-7 and MDA-MB-231 cells by SCS alone and in combination with tamoxifen.** Cells were stained with FLICA™ FAM-VAD-FMK for detection of active caspase-9 and labeled with PI and Hoescht stain following treatment with SCS (8.5 and 10.0 μg/ml for MCF-7 and MDA-MB-231 cells, respectively), tamoxifen (5.0 μM) or their combination for 24 h. Caspase-FLICA stains active caspases green; PI stains dead or dying cells red; Hoescht stains the nuclei of all cells blue.

Prior work by our group demonstrated that SCS activated the effector caspase 3/7 in both MCF-7 and MDA-MB-231 cells [13]. In the current study, we observed that the initiator caspases 8 and 9 were also strongly activated by SCS in both cell lines as indicated by intense green fluorescence signal that directly reflects the amount of caspase activity in the cells (Figures 7 and 8). Tamoxifen on the other hand, activated these caspases in a smaller population of MCF-7 and MDA-MB-231 cells. When these cells were co-treated with both agents, high activation of both caspases was observed.

### Cytotoxic acitivity and DNA damage in non-cancerous MCF10A cells

**Figure 9:**
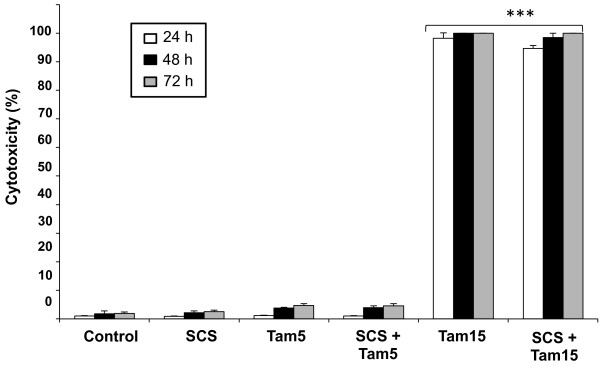
**Cytotoxic effects of SCS alone and in combination with tamoxifen on MCF-10A cells.** Cytotoxicity was measured at 24, 48 and 72 h of treatment with SCS (8.5 μg/ml), tamoxifen (5 and 15 μM) and their combination using the LDH assay. DMSO was used as a vehicle control. Data shown are the mean values ± SD from three independent experiments. Statistical analysis was determined using one-way ANOVA followed by post hoc Tukey multiple comparison test with *p < 0.05 and ***p < 0.001 compared to control.

**Figure 10:**
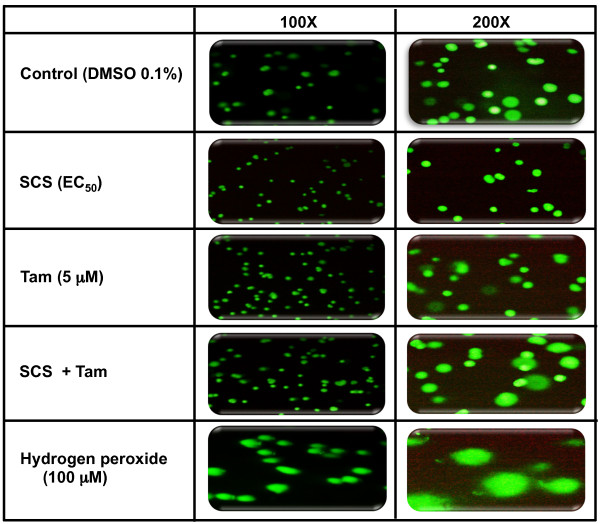
**Effect of SCS alone and in combination with tamoxifen on the DNA integrity of MCF-10A cells.** Cells were treated with SCS (8.5 μg/ml), tamoxifen (15 μM) and their combination for 24 h and stained with SYBR Green for evaluation of DNA damage using the Comet Assay. H_2_O_2_ (100 μM) was used as the positive control. The micrographs show images of DNA captured under fluorescence microscopy. Cells with damaged DNA display comet tails while undamaged DNA will appear as intact DNA head.

Since SCS could promote the effects of tamoxifen on MCF-7 and MDA-MB-231 cells, we investigated whether a similar response would occur in the non-cancerous breast epithelial cells, MCF-10A. A small but insignificant increase in the percentage of cell death was observed with 5.0 μM tamoxifen treatment compared to untreated cells, while 15 μM tamoxifen was found to be highly cytotoxic (Figure 9). SCS however, was not cytotoxic to the cells and did not significantly modulate tamoxifen-induced cytotoxicity, unlike the observations with the cancer cells above. In addition, analysis using the Comet assay (also called single cell gel electrophoresis) further showed that the DNA integrity of MCF-10A cells was not compromised by SCS treatment (Figure 10). Cells with damaged DNA would display DNA comet tails that consist of single-stranded or double-stranded DNA breaks. Tamoxifen (5.0 μM) treatment showed the presence of some DNA damage with a few comet tails detected and the presence of SCS did not significantly modulate the tamoxifen effect. Exposure of MCF-10A cells to H_2_O_2_ showed extensive DNA damage.

## Discussion

The *S. crispus* plant has been traditionally used since time immemorial and has been of much interest due to traditional claims of its anticancer properties [6]. Its widespread use is evidenced by its reference in the publication of the Indonesian pharmaceutical regulatory agency [7]. Based on our previous findings of apoptotic cell death induced by a bioactive subfraction of the plant, SCS, the present study was carried out to examine possible synergistic interaction between SCS and tamoxifen in inhibiting growth and inducing death of breast cancer cells. Importantly, evaluation on the non-cancerous breast epithelial cell line, MCF-10A, was also performed to provide additional information on the potential use of this natural product in the clinical setting.

Tamoxifen is a partial agonist of ER, blocking the proliferative effects of estrogen via this receptor. It causes growth arrest at nanomolar concentrations but cell death at micromolar concentrations [16]. In the current study, 2.5 to 15.0 μM tamoxifen were tested on MCF-7 and MDA-MB-231 cells. Clinically relevant steady-state plasma concentrations of tamoxifen and its metabolites can be close to 5 μM in the patient sera [17] and 5 – 11 times higher in intratumoral tissues such as the mammary gland [18]. Thus the concentrations used in this study are within the range achievable in human plasma.

Combination therapy offers the advantage of possible dose reduction via synergistic growth inhibition of cancerous cells with potential reduction in toxicity caused by the chemotherapeutic agent. We demonstrated for the first time that a subfraction of *S. crispus* synergistically promoted tamoxifen-induced cytotoxicty of breast cancer cells. Induction of cell death was time- and dose-dependent, with near maximum cell death achieved when combined with 2.5 μM tamoxifen, suggesting the potential of using a lower tamoxifen dose to achieve the same level of effectiveness as demonstrated by a high tamoxifen dose alone. This may supposedly also reduce the toxicity and perhaps inhibit the development of resistance to this drug.

In line with this notion, SCS increased the level of apoptosis induced by tamoxifen in both MCF-7 and MDA-MB-231 cell lines. Similar levels of apoptosis were achieved when SCS was combined with either 2.5 μM or 5.0 μM tamoxifen after 48 h exposure, further indicating that SCS synergises with tamoxifen to achieve efficacy at lower drug concentrations in both cell lines. In addition, SCS enhanced the rate of apoptosis in both cell lines with higher percentages of cells in late stage apoptosis compared to tamoxifen alone. It is noteworthy that tamoxifen induced a lower percentage of apoptosis in MDA-MB-231 cells compared to MCF-7 cells which is consistent with the findings of Kallio et al. [19] who reported a slower death rate in the ERα-negative cells by this drug.

Mitochondrial dysfunction is considered as an early event in the apoptotic cascade, characterised by an increase in membrane permeability and loss of ΔΨ_m_ (reviewed in [20]). Mitochondria of cancer cells display higher ΔΨ_m_[21] and chemotherapeutic agents induce mitochondrial membrane permeabilization resulting in the collapse of ΔΨ_m_[22]. In the present study, tamoxifen-induced apoptosis of MCF-7 and MDA-MB-231 cells was accompanied by dose-dependent depolarization of the mitochondrial membrane, similar to earlier findings [19]. SCS seems to be more potent in promoting membrane permeabilization in these cells. Further depolarization of the mitochondrial membrane occurred when SCS was combined with tamoxifen. Kallio et al. [19] reported 7 μM as the threshold concentration of tamoxifen needed to induce the mitochondrial pathway. Our data show that 80% or more reduction in the ΔΨ_m_ of MCF-7 cells is achieved with only 2.5 μM tamoxifen in the presence of SCS, indicating the ability of SCS to sensitize the cancer cells to mitochondrial membrane disruption. Similar effects were noted in MDA-MB-231 cells suggesting an ERα-independent action. Loss of mitochondrial membrane potential would lead to cytochrome c release from the intermembrane compartment of mitochondria to the cytosol [23].

Caspases are responsible, in part, for the cellular changes during apoptosis. They are synthesised as zymogens (procaspases) that are activated by proteolytic cleavage and can cleave many cellular substrates in regulating apoptosis [24]. Intrinsic and extrinsic pathways are two established mechanisms for caspase-dependent apoptosis. The intrinsic pathway transduces cell death signals via the mitochondrial system while the extrinsic pathway involves ligand binding to death receptor complexes on cell surfaces. Both events involve activation of initiator caspases, leading to cleavage and activation of effector caspases to execute apoptotic cell death (reviewed in [25]).

We previously demonstrated that SCS induced apoptosis of both MCF-7 and MDA-MB-231 cells by activating the executioner caspase-3/7 [13]. MCF-7 cells are functionally deficient of caspase-3 [26] and thus, effector caspase-7 mediates apoptosis of these cells [27]. Here we show that SCS strongly activates initiator caspase-8 in both cells while tamoxifen did not significantly activate it in MCF-7 cells. Caspase-8 plays a role in the extrinsic apoptosis pathway and is activated following stimulation of cell surface death receptors such as DR, Fas and tumour necrosis factor-related (TNFR) death receptors or activation of their corresponding ligands. This leads to the formation of death-inducing signalling complex (DISC) resulting in proteolytic activation of procaspase-3/7 [25]. Stimulation with chemotherapeutic agents are reported to cause caspase-8 activation which directly activates caspase-3 [22,28] or in the case of MCF-7 cells, caspase 7, leading to apoptosis. We observed that co-treatment of both MCF-7 and MDA-MB-231 cells with tamoxifen and SCS caused strong activation of caspase-8, in line with the synergistic cytotoxic effect and the promotion of apoptotic cell death above. This could be contributed by the presence of β-sitosterol, one of the identified components of *S. crispus*[11], reported to induce apoptosis of MDA-MB-231 cells [29] and increase caspase-8 activity in MCF-7 and MDA-MB-231 cells in association with elevation of Fas protein expression [30].

Alternatively, caspase-8 cleaves BH3-only protein, Bid, to generate the truncated form of Bid, t-Bid [31]. Translocation of t-Bid to the mitochondrial outer membrane promotes the release of cytochrome c that binds to Apaf-1 adaptor protein to form apoptosome with the recruitment and activation of caspase-9, a key initiator caspase of the intrinsic pathway. Activation of caspase-9 following caspase-8 activation has been reported, leading to the activation of the effector caspase-3 [32]. Although a previous study did not show caspase-9 cleavage in MCF-7 cells [19], we observed some caspase-9 activity in both MCF-7 and MDA-MB-231 cells with similar concentrations of tamoxifen. Interestingly, SCS strongly activates caspase-9 in both MCF-7 and MDA-MB-231 cells either alone or in the presence of tamoxifen, indicating that the SCS is also capable of stimulating the intrinsic pathway of apoptosis. In agreement to this, a recent study reported that *S. crispus* ethanol extract increased caspase-9 concentration and induced cytosolic translocation of cytochrome c in MCF-7 cells [33].

Other studies have also reported enhanced activities of chemotherapeutic drugs by plant-derived products. Examples include the synergistic inhibition of HT-29 colon cancer cell growth by the chemotherapeutic drug 5-fluorouracil (5-FU) and genistein (soy flavone) combination, which involves upregulation of pro-apoptotic p53 and p21 [34]. Gambogic acid (a component of resin from Garcinia hanburryi tree) is also able to enhance the sensitivity of BGC-823 gastric carcinoma cells to 5-FU by regulating key enzymes in the 5-FU metabolic pathway [35]. Recently, curcumin (yellow pigment of turmeric), which acts as an inhibitor of cyclooxygenase-2 was demonstrated to enhance the growth inhibitor effects of 5-FU on HT-29 cells [36].

Studies on the combination of tamoxifen with other agents such as TRAIL [37] and rapamycin [38] have been reported to produce synergistic apoptotic activity. However, the therapeutic potential of TRAIL for example, is limited by concerns over its potential hepatotoxicity [39,40]. Tamoxifen itself was found to be more cytotoxic to normal cells compared to cancerous cells [41]. This was thought to be due to higher expression of ER in the normal cells. Unlike tamoxifen, SCS is non-cytotoxic to the non-cancerous MCF-10A cells [13]. In addition, contrary to our observation with the breast cancer cells above, SCS does not potentiate the cytotoxic effect of tamoxifen on MCF-10A cells. SCS also does not promote DNA damage either as a single agent or in combination with tamoxifen. This is an important observation since tamoxifen is capable of forming DNA adducts in treated women [42,43]. Tamoxifen treatment of breast cancer patients or as a chemopreventive agent has in fact been associated with increased risk of endometrial cancer [44,45] thought to be due to its estrogenic activity on the endometrium via ER or as a result of tamoxifen-DNA adduct formation [46].

## Conclusion

An exciting outcome from this study is the observation that SCS synergized the cytotoxic action of tamoxifen at sub-optimal doses of the drug not only in ERα-positive MCF-7 but also in the ERα-negative MDA-MB-231 cells. The fact that we can use sub-optimal doses of tamoxifen to achieve the desired cytotoxic effect suggests the potential for reduction in side effects/toxicity as observed on non-cancerous cells (as depicted in MCF-10A). The possibility of SCS to act via a mechanism independent of ER thus provides the opportunity to target multiple cancer signaling pathways when combined with the anti-estrogen. Although cell death is controlled by multiple inputs, the mitochondria play a central role in its major pathway and our findings show enhancement of apoptosis via perturbation of mitochondrial function and activation of caspases in both intrinsic and extrinsic pathways. The mechanism is likely to be dependent on transcriptional regulation of apoptotic signaling proteins. Investigation on the contribution of these signaling proteins and the potential of SCS to overcome or prevent resistance to tamoxifen can be the focus of future studies. The presence of multiple compounds in natural products such as SCS can provide the advantage of acting on multiple pathways that control the process of cancer development and progression. The potential of the *S. crispus* subfraction is thus to be exploited for improvement of therapeutic responses and perhaps the reduction in drug toxicity in cancer therapy.

## Abbreviations

SCS: *Strobilanthes crispus* subfraction; ER: Estrogen receptor; LDH: Lactate dehydrogenase; CI: Combination index; ∆Ψ_m_: Mitochondrial membrane potential; JC-1: 5,5′,6,6′-tetrachloro-1,1′,3,3′-tetraethyl-benzimidazolylcarbocyanine iodide; PI: Propidium iodide.

## Competing interests

The authors declare that they have no competing interests.

## Authors’ contributions

NSY conceived and designed the study, interpreted the data and prepared the manuscript. NNNMK performed the experimental work, prepared and analysed the data and participated in the preparation of the manuscript. MNN participated in the study design and preparation of the manuscript. All authors read and approved the final manuscript.

## Pre-publication history

The pre-publication history for this paper can be accessed here:

http://www.biomedcentral.com/1472-6882/14/252/prepub
